# The capacity and training needs of primary health care workers in Nyeri and Nakuru counties of Kenya: a cross-sectional study

**DOI:** 10.3389/fmed.2024.1466383

**Published:** 2025-01-07

**Authors:** Josphat Martin Muchangi, Kioko Kithuki, Sarah Jebet Kosgei, Mary Mathenge, Deborah Kioko, Bryson Sifuma, Billian Sawenja, Samuel Kamau

**Affiliations:** ^1^AMREF Health Africa, Nairobi, Kenya; ^2^Reinit Research, Nairobi, Kenya

**Keywords:** health services, competence, health system, primary healthcare, health personnel

## Abstract

**Background:**

Health systems based on primary healthcare (PHC) have reduced costs and are effective for improved health outcomes. Kenya’s health system grapples with providing equitable access to essential health services, but there is increasing commitment by the government to strengthen primary healthcare. The aim of this paper is to provide a baseline assessment of the capacity and training needs of healthcare workers (HCWs) in Nakuru and Nyeri Counties and identify priorities for intervention.

**Methods:**

A cross-sectional study was carried out among 171 healthcare workers in Nyeri and Nakuru counties. Multistage sampling was employed to select sub-counties in the first stage and health facilities by level within each sub-county in the second level. Systematic random sampling was then employed to select HCWs from each level of facility. We targeted healthcare workers of all cadres within the health facilities, and included all who consented to participating. Structured self-administered pen-and-paper questionnaires were used for data collection, and a five-point Likert scale was used to measure the perceived capacity of the healthcare workers to provide primary healthcare. As for the training needs data, the participants selected any of the 12 components that they needed training in. Descriptive statistics was employed, and stacked bar charts were used to visualize the capacity and training needs for the components of PHC adopted in Kenya.

**Results:**

In total, we obtained questionnaires from 95 participants in Nakuru and 76 participants in Nyeri. Nakuru HCWs rated themselves higher than their Nyeri counterparts in maternal and newborn child healthcare, local endemic disease control, appropriate treatment of common diseases and injuries, provision of essential basic medication, dental health, HIV/AIDs & TB management, and primary eye care. In both counties, there were significant differences in capacity between the different levels of health facilities. We observed substantial capacity gaps for HIV/AIDs & TB management, mental health and dental health services in both counties.

**Conclusion:**

This study found a substantial capacity gap in several of the elements of PHC, especially in Nyeri County. Critical areas for intervention are HIV/AIDs & TB management and mental health training for both counties. Within the health system, there is need to strengthen the capacity of HCWs in lower-level health facilities to reduce the volume of referrals to secondary care facilities. We strongly recommend training programs in dental health, mental health, primary eye care, nutritional services and HIV/AIDs &TB management, that are carefully designed to emphasize skills and abilities.

## 1 Introduction

Evidence abounds on the association of primary healthcare and well-functioning health systems. Studies report on lower hospitalization risks, healthcare costs, and infant mortality rates associations with strengthened primary healthcare (1–4). Health systems based on primary healthcare (PHC) attain better health outcomes than those reliant on a vertical mode of health services delivery. The Alma Ata declaration highlighted eight elements to implement PHC in health systems: health education for prevention and control of disease, effective food supply and proper nutrition, maternal and child healthcare (including family planning); adequate water, sanitation and hygiene services, immunization against major infectious diseases, local endemic diseases control, appropriate treatment of common diseases and injuries and provision of essential medication (5). The Kenyan government’s primary care strategic framework adds dental health, mental health, HIV/AIDS, and primary eye care to ensure quality PHC in the Kenyan context (6). The government also adopted PHC as the linchpin of its commitment to attain Universal Health Coverage by 2022. However, the current PHC system is yet to fulfil its potential.

The health system in Kenya grapples with delivering equitable access to essential health services, especially to geographically diverse counties. The devolved governance structure places responsibility for healthcare budgets and facilities on individual counties. This approach can lead to disparities, with urban areas typically boasting more facilities and specialists compared to remote villages (7). The mix of nurses, doctors, and community health workers varies across counties (8). Kenya falls short of the WHO’s recommended ratios, hindering the ability to deliver optimal care (9). The situation is further complicated by rapid urbanization in some Counties, straining existing resources. This leads to a vicious cycle, where patients bypass PHC facilities due to poor quality services and pay out of pocket for higher-level specialist services. This lowers the influx of funds to PHC facilities, thus intensifying the problems undermining PHC.

Nakuru county had a total population of 2,162,202 and a population density of 290 people per sq. Km while Nyeri county had a population of 759,164 and a population density of 228 people per sq. Km. according to the 2019 census (10). Nakuru had a median age of 19 years, while Nyeri had a meadian age of 20 years (11). The healthcare systems in Nakuru and Nyeri Counties have common characteristics. Both counties grapple with delivering healthcare services across urban centers and remote villages. Both fall below the WHO-recommended health worker ratios (12, 13). Despite prioritizing PHC, both counties face a high burden of preventable diseases like respiratory infections and diarrhea (14). Both counties allocate a significant portion of their budgets to healthcare compared to other Kenyan counties, indicating a strong commitment to improvement. The willingness of the county health management teams in both Nakuru and Nyeri to collaborate with partners suggests a proactive approach to addressing healthcare challenges. There are also some key differences. Nakuru is experiencing rapid urbanization, straining existing facilities despite the potential benefit of a concentrated population. Nyeri, on the other hand, remains predominantly rural. Additionally, the specific mix of nurses, doctors, and community health workers might differ between the counties.

These factors present a unique opportunity for investigations to guide the implementation of reforms by the government and development partners amidst diverse challenges. This paper provides a baseline assessment of the capacity and training needs of healthcare workers (HCWs) in Nakuru and Nyeri Counties and identifies intervention priorities. The findings will inform training activities to match the knowledge, ability, and skills of the healthcare workers to the needs of the respective communities.

## 2 Materials and methods

### 2.1 Study design

**Figure 1:**
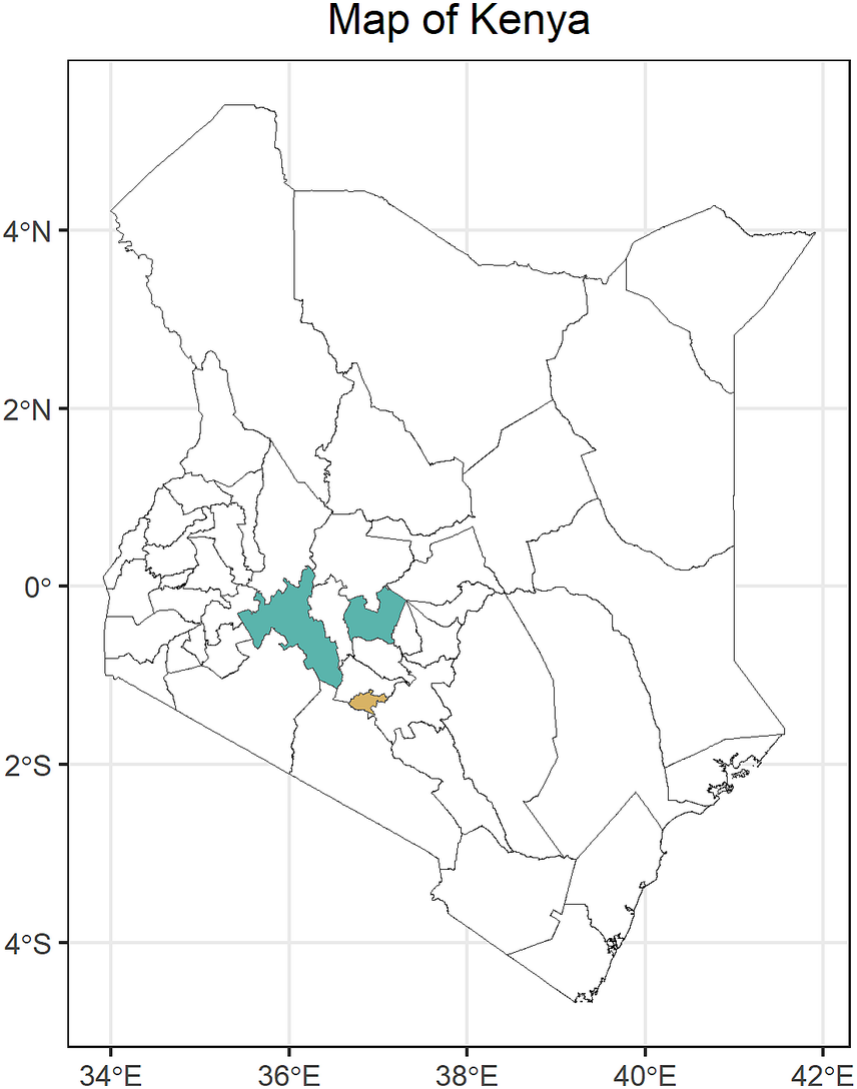
Map of Kenya showing the geographical position of the study areas, Nakuru and Nyeri counties from left to right. The geographic position of Nairobi County is shown.

A cross-sectional study was carried out among healthcare workers in the two counties. This study was carried out in four sub-counties within Nakuru County and three sub-counties within Nyeri County, Kenya (Figure 1).

### 2.2 Sampling strategy

For the HCWs survey, the sample size was determined separately for each of the two counties given their difference in characteristics. The Cochrane formula was used to calculate the sample size. A confidence interval of 95% and a margin of error of 5% was applied. The proportion of the variables of interest was assumed at 50% (to give maximum variability). The calculated sample size was 378, and by adding a 10% non-response rate, the final sample size was 416. Since the population of HCWs is known, a finite population correction was applied. The required sample size from Nakuru was 89, and that from Nyeri was 65.

We used multistage sampling to identify and select assessment participants. In the first stage, we selected four sub-counties in Nakuru and three sub-counties in Nyeri. All seven sub-counties above were sampled to ensure the total number of health facilities was sufficient to generalize the findings to the counties. In the second stage, public health facilities were randomly selected by level within the selected sub-counties from the Kenya Health Information System (15). Level 2 facilities consisted of clinics and dispensaries, level 3 consisted of health centers and level 4 facilities consisted of sub-county hospitals, as per Kenyan health system definitions (16). We applied probability proportionate to size sampling, meaning a sub-county with a higher population of health facilities was allocated more respondents. In the third stage, systematic random sampling was used to determine the participating healthcare workers within each health facility. Within a health facility, the enumerator interviewed the facility head or a healthcare worker in charge of PHC that was available.

Respondents for Key Informant Interviews were selected through a purposive sampling technique that involved identifying and selecting individuals or groups of individuals that were especially knowledgeable about or experienced on the PHC delivery. For each county, we targeted the County Health Management Team (CHMT) as well as the Sub-County health management teams (SCHMT) as well as the National PHC Technical Advisor within the Ministry of Health, and the Project Manager of Project THRIVE, under which this study is conducted. The CHMT consisted of County Records Health Information Officers, County Primary Care Coordinators and County Community Strategy Focal Persons. The SCHMT consist Sub-county Public Health Nurses, Sub-county Public Health Officers, Sub-county Records Health Information Officers.

### 2.3 Eligibility criteria

All healthcare workers who had worked in targeted health facilities within the chosen sub-counties for at least 3 months and gave informed consent were included. As for the key informants, only those who had the authority to speak on behalf of the government agency they represent and gave informed consent to participate were included.

### 2.4 Data collection, handling and analysis

A structured questionnaire was developed for data collection, including demographic information and questions related to the capacity of the HCWs, and their perceived training needs. The demographic information included participants’ county, sub-county, name of the facility, level of the facility, job title, duration of years of working/clinical experience, current department within the facility, and working years at the facility. For the capacity self-evaluations, the participants rated their competence on the eight components of primary healthcare in addition to four other components: primary eye care, HIV/AIDs and TB management, mental health, and dental health. We employed a 5-point Likert scale, with 1 = ‘Not confident’ and 5 = ‘Very confident’ for the ratings. As for the training needs data, the participants selected any of the 12 components that they needed training in.

The HCWs’ survey questionnaires were programmed into a digital format using the KoBoCollect. The digital tools were downloaded on smartphones that enumerators used to collect data. The digital questionnaire had quality control features, including in-built skip logic, mandatory inputs, consistency checks, and a Global Positioning System (GPS) to enable geospatial mapping of the surveyed households.

For structured questionnaires, interviewers uploaded completed instruments to the server at the end of each working day, allowing the investigators to review data entries daily. The data were exported into RStudio v.4.4.0 for cleaning, following the data quality assurance strategy and then analysis. Descriptive statistics, such as frequency, percentage, mean, and standard deviation, were performed. A *p*-value of 0.05 was used as the threshold of statistical significance. We assessed whether assumptions of normal distribution (by Kolmogorov–Smirnov test) were met before further analysis. We found that the data were not normally distributed and used the Mann–Whitney *U* test and Kruskal Wallis H-test to analyze differences in mean confidence ratings of the 13 components between the two counties, and at different facility levels. Dunn’s test with Bonferroni adjustment was used as a post-hoc analysis for results with significant differences in the Kruskal Wallis test step.

### 2.5 Ethical considerations

Ethical approval was obtained from the University of Eastern Africa, Baraton University Research Ethics Committee. A standardized informed consent form was used to request and secure the informed consent. Respondents who obliged after being informed were asked to sign or apply their thumbprint on the consent forms. Confidentiality was maintained by not requiring the respondents to provide their names. Digital files were protected using passwords, while only authorized personnel had access to hard copies of the study materials. The data collection teams were trained in confidentiality and privacy.

## 3 Results

### 3.1 Respondents characteristics

A total of 171 HCWs met the inclusion criteria and participated in the survey. Over half of the study participants were from Nakuru County. The majority work in Level 2 facilities, followed by Level 3 facilities. Level 4 facility workers make up the least proportion.

Nurses were the most prevalent cadre, accounting for over two-thirds of respondents. Clinic Officers comprised a notable portion (11%) followed by Public Health Officers (4%). Less prevalent cadres included: Nutritionists, Lab technicians, Pharmacist, Doctors, and other healthcare workers, e.g., Radiologists, Occupational therapists, and Health Records Information Officers. The median years of experience in the current role were 9 and 13 years for Nakuru and Nyeri Counties, respectively. 71% of HCWs in Nakuru and 39% of their Nyeri counterparts had worked at their current facility for less than 5 years. Most of the respondents worked in the Outpatient Department (OPD), suggesting that OPD services are a focus in many of the facilities in the survey. Maternal Child Health (MCH) represents the second most common section. A substantial proportion of respondents work in Pharmacy, highlighting the importance of pharmaceutical services in the healthcare delivery system. Table 1 gives a summary of the respondents’ characteristics.

**Table 1:** County, level of facility, cadre and department of the healthcare workers who participated in the survey.

Description	Nakuru (*N* = 95)	Nyeri (*N* = 76)
Level of facility		
Level 2	53 (56%)	43 (57%)
Level 3	24 (25%)	22 (29%)
Level 4	18 (19%)	11 (14%)
Cadre		
Nurse	73 (76%)	51 (67%)
Clinic officer	8 (8%)	11 (14%)
Other	6 (6%)	10 (13%)
Public health officer	6 (6%)	1 (1%)
Nutritionist	2 (2%)	3 (4%)
Doctor	1 (1%)	0
Median years of experience in current role (years)	9	13
Department		
OPD	73 (77%)	67 (88%)
MCH	60 (63%)	52 (68%)
Pharmacy	32 (34%)	47 (62%)
Maternity	33 (35%)	13 (17%)
Health records	11 (12%)	27 (36%)
Comprehensive care	13 (14%)	11 (14%)
Other	18 (19%)	2 (3%)
Casualty & emergency	8 (8%)	12 (16%)
In patient	7 (7%)	9 (12%)
Lab & blood bank	10 (11%)	6 (8%)
Public health	13 (14%)	1 (1%)
Median years of experience in current facility (years)	3	5

### 3.2 Status of capacity to provide primary healthcare among the HCWs

#### 3.2.1 Comparison of mean confidence ratings between Nakuru and Nyeri counties

**Figure 2:**
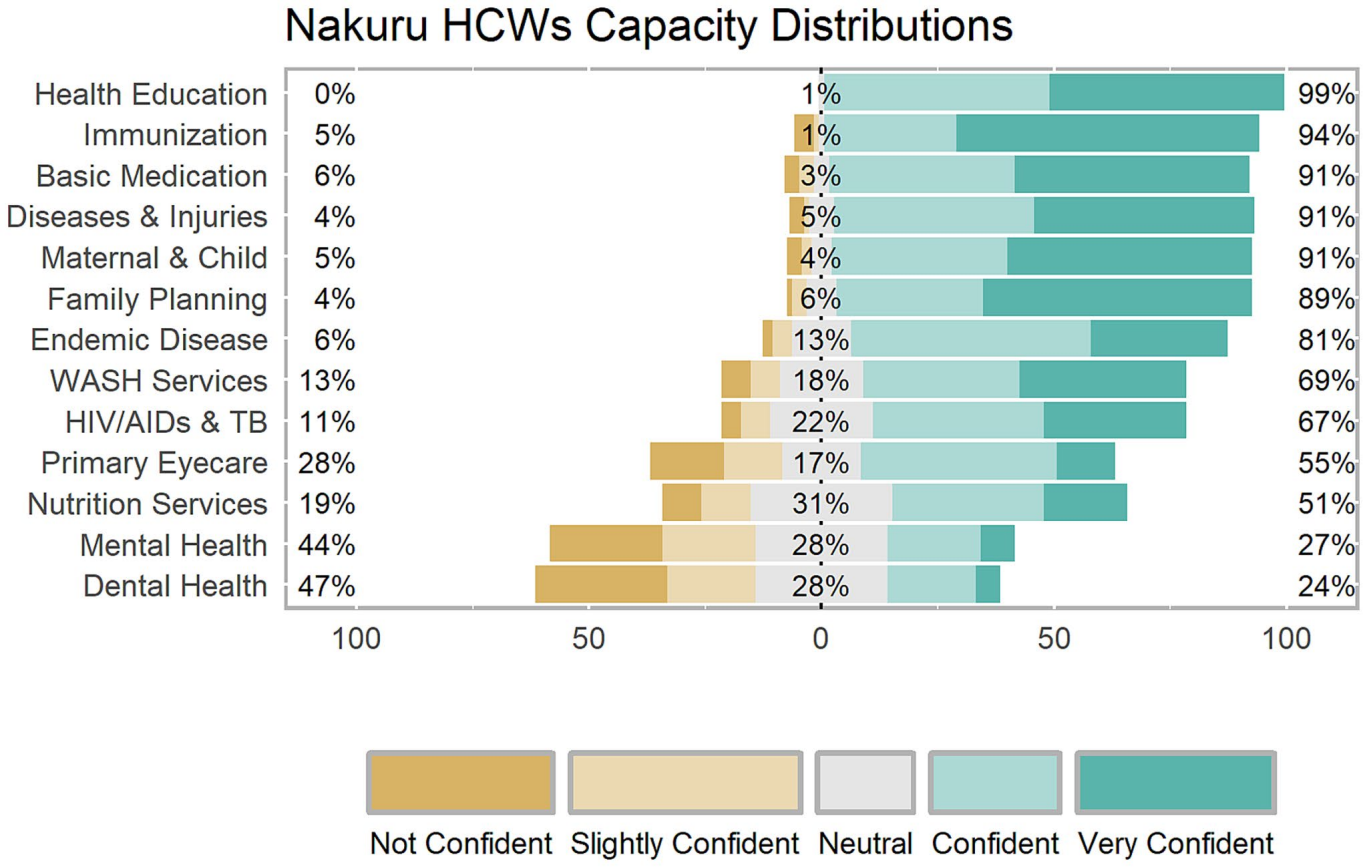
Likert plot of capacity levels of healthcare workers in Nakuru County.

**Figure 3:**
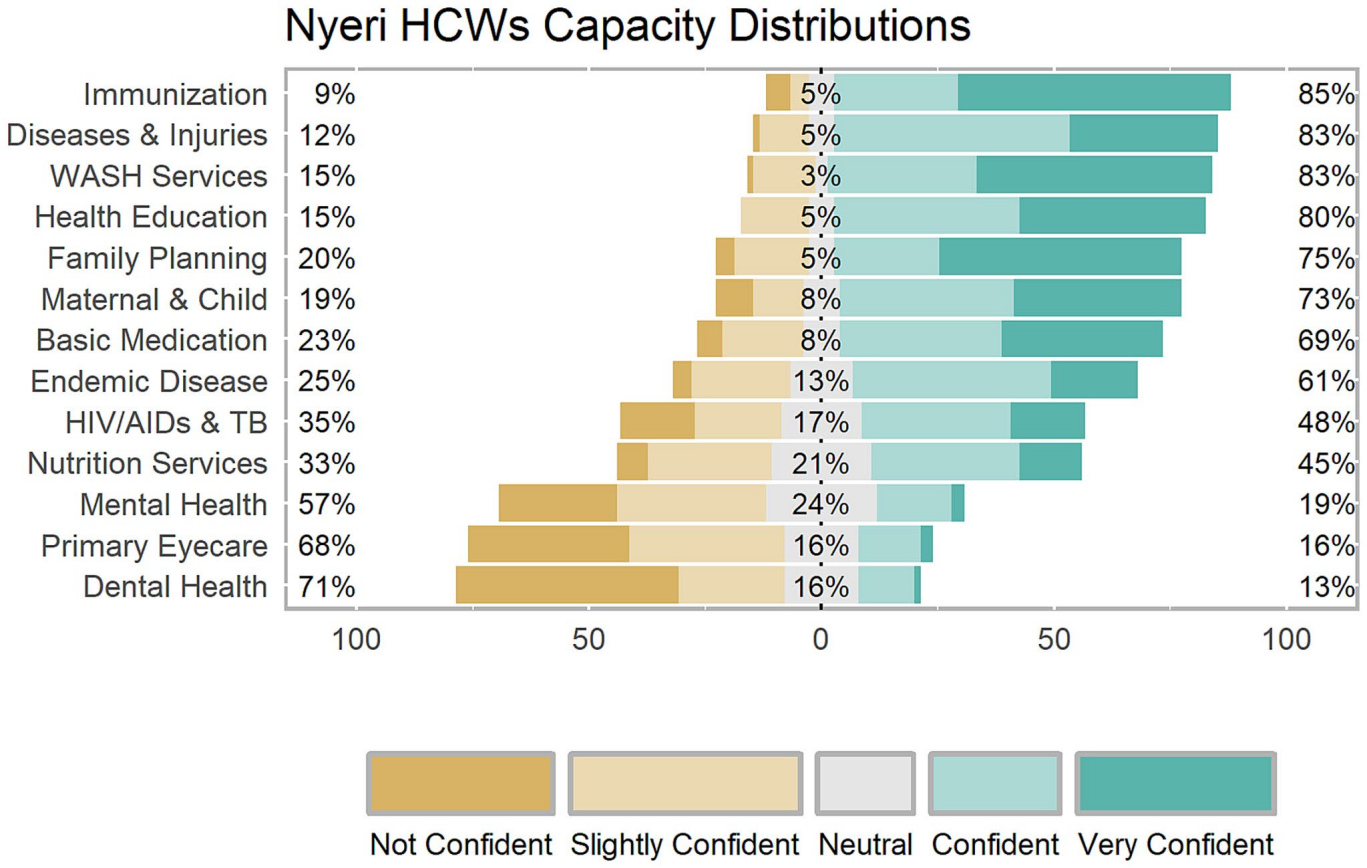
Likert plot of capacity levels of healthcare workers in Nyeri County.

The HCWs ratings revealed high confidence in several core components of primary healthcare. However, they demonstrated low confidence in their capacity to provide dental health and mental health services (Figures 2, 3).

The mean ratings among HCWs in Nakuru ranged from (2.55 ~ 4.50), while those in Nyeri ranged between (1.95 ~ 4.30). Table 2 demonstrates the mean of total confidence ratings of workers in the two Counties on the different components of primary healthcare investigated.

**Table 2:** Perceived capacity levels of healthcare workers in Nakuru and Nyeri counties.

County	Nakuru (*N* = 94)	Nyeri (*N* = 76)
Components of primary healthcare	Mean (SD)	Mean (SD)
Education on health problems and how to prevent and control them.	4.50 (0.52)	4.05 (1.02) *
Development of adequate food supply and proper nutrition	3.44 (1.13)	3.16 (1.19)
Maternal and newborn child healthcare	4.38 (0.84)	3.79 (1.29) **
Family planning	4.42 (0.84)	4.03 (1.25)
Adequate and safe water supply and basic sanitation	3.89 (1.13)	4.13 (1.14)
Immunization against major infectious diseases	4.49 (0.92)	4.30 (1.10)
Local endemic disease control	4.01 (0.89)	3.53 (1.15) **
Appropriate treatment of common diseases and injuries	4.29 (0.88)	4.03 (0.97) *
Provision of essential basic medication	4.35 (0.86)	3.72 (1.28) **
Dental Health	2.55 (1.23)	1.95 (1.12) **
Mental Health	2.67 (1.25)	2.38 (1.11)
HIV/AIDS & TB management	3.83 (1.07)	3.14 (1.33) ***
Primary eye care	3.26 (1.27)	2.14 (1.13) ****

(*p < 0.05, **p < 0.01, ***p < 0.001, *p* < 0.0001* for Mann Whitney *U* test comparison of mean confidence ratings, where alpha = 0.05).

Notably, Mann–Whitney *U* test results revealed statistically significant differences in capacity levels between HCWs in the two counties. Nakuru healthcare workers rated themselves higher than their Nyeri counterparts in maternal and newborn child healthcare, local endemic disease control, appropriate treatment of common diseases and injuries, provision of essential basic medication, dental health, HIV/AIDs & TB management, and primary eye care.

#### 3.2.2 Comparison of confidence ratings among HCWs in different level facilities

In Nakuru, Level 2 HCWs rated themselves significantly higher than Level 4 HCWs for family planning healthcare services. Level 2 HCWs rated themselves significantly lower than their Level 4 counterparts in providing HIV/AIDs & TB management healthcare. There were no significant differences between facility levels in all other elements of primary healthcare. Table 3 gives a comparison of mean ratings between the health facility levels in Nakuru.

**Table 3:** Comparison of self-rated confidence levels between the three health facility levels in Nakuru county.

Nakuru county			
Facility level	Level 2 (*N* = 53)	Level 3 (*N* = 24)	Level 4 (*N* = 18)
Components of primary healthcare	Mean (SD)	Mean (SD)	Mean (SD)
Education on health problems and how to prevent and control them	4.51 (0.50)	4.42 (0.50)	4.56 (0.62)
Development of adequate food supply and proper nutrition	3.19 (1.13)	3.63 (1.24)	3.89 (0.76)
Maternal and newborn child healthcare	4.43 (0.77)	4.21 (1.02)	4.50 (0.79)
Family planning	4.66 (0.68)*	4.29 (0.86)	3.94 (1.00)*
Adequate and safe water supply and basic sanitation	3.79 (1.04)	4.13 (1.19)	3.83 (1.29)
Immunization against major infectious diseases	4.60 (0.84)	4.50 (0.93)	4.12 (1.11)
Local endemic disease control	3.87 (0.92)	4.17 (0.76)	4.22 (0.88)
Appropriate treatment of common diseases and injuries	4.34 (0.73)	4.08 (1.21)	4.44 (0.70)
Provision of essential basic medication	4.38 (0.81)	4.21 (1.02)	4.39 (0.85)
Dental Health	2.30 (1.10)	2.88 (1.36)	2.89 (1.28)
Mental Health	2.40 (1.13)	3.13 (1.30)	2.89 (1.37)
HIV/AIDS & TB management	3.51 (1.07)*	4.21 (0.66)*	4.28 (1.23)*
Primary eye care	3.13 (1.30)	3.38 (1.31)	3.44 (1.10)

*p < 0.05, Indicates that the result is statistically significant at the 5% significance level.

In Nyeri, Level 3 HCWs rated themselves significantly lower for immunization against major diseases than Level 2 and 4 HCWs. Collectively and at different facility levels, Nyeri healthcare workers demonstrated critically low confidence in providing dental healthcare services. Level 2 and 3 HCWs had significantly lower mean confidence ratings than level 4 HCWs, who had a mean of 3.45 for this component. Concerning mental healthcare and primary eyecare, level 4 HCWs rated their confidence levels significantly higher than their counterparts in lower-level facilities, especially Level 2 facilities. Table 4 gives a comparison of mean ratings between the health facility levels in Nyeri.

**Table 4:** Comparison of self-rated confidence levels between the three health facility levels in Nyeri county.

Nyeri county			
Facility level	Level 2 (*N* = 43)	Level 3 (*N* = 22)	Level 4 (*N* = 11)
Components of primary healthcare	Mean (SD)	Mean (SD)	Mean (SD)
Education on health problems and how to prevent and control them.	4.12 (1.05)	4.00 (1.07)	3.91 (1.07)
Development of adequate food supply and proper nutrition	3.14 (1.32)	2.86 (0.94)	3.82 (0.94)
Maternal and newborn child healthcare	3.77 (1.34)	3.68 (1.36)	4.09 (1.36)
Family planning	4.05 (1.23)	3.77 (1.48)	4.45 (1.48)
Adequate and safe water supply and basic sanitation	4.16 (1.19)	3.95 (1.13)	4.36 (1.13)
Immunization against major infectious diseases	4.58 (0.88)	3.77 (1.45)*	4.27 (1.45)
Local endemic disease control	3.58 (1.10)	3.41 (1.22)	3.55 (1.22)
Appropriate treatment of common diseases and injuries	4.02 (0.94)	3.86 (1.17)	4.36 (1.17)
Provision of essential basic medication	3.95 (1.17)	3.27 (1.58)	3.73 (1.58)
Dental Health	1.74 (0.90)	1.59 (0.91)	3.45 (0.91)*
Mental Health	2.16 (1.00)	2.27 (1.08)	3.45 (1.08)*
HIV/AIDS & TB management	2.84 (1.25)	3.14 (1.42)	4.36 (1.42)
Primary eye care	2.28 (1.08)	1.95 (1.09)	2.00 (1.09)

*p < 0.05, Indicates that the result is statistically significant at the 5% significance level.

### 3.3 Training needs identified by the HCWs

**Figure 4:**
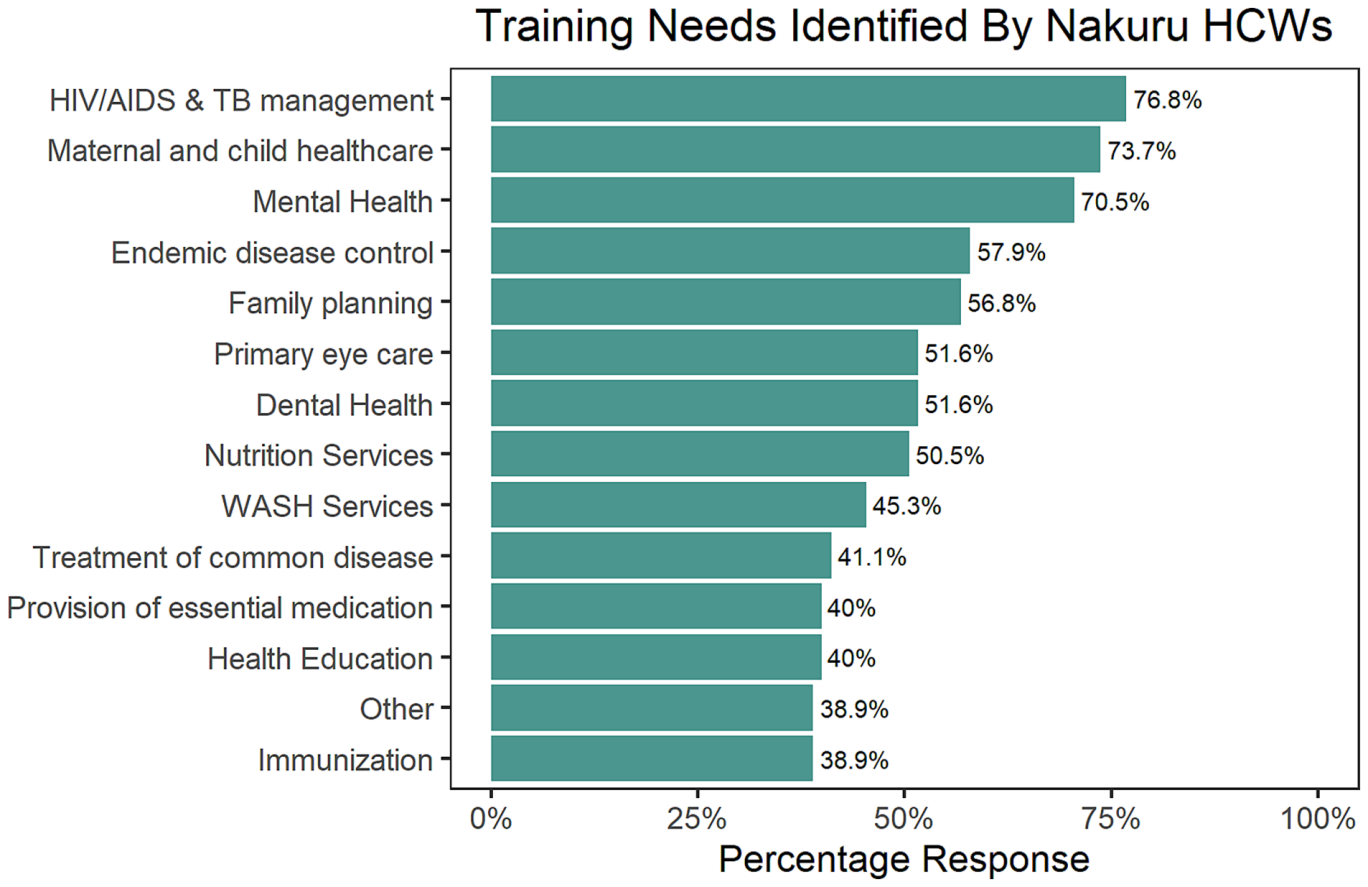
The training needs identified by Nakuru County HCWs, ranked in order of percentage frequency.

**Figure 5:**
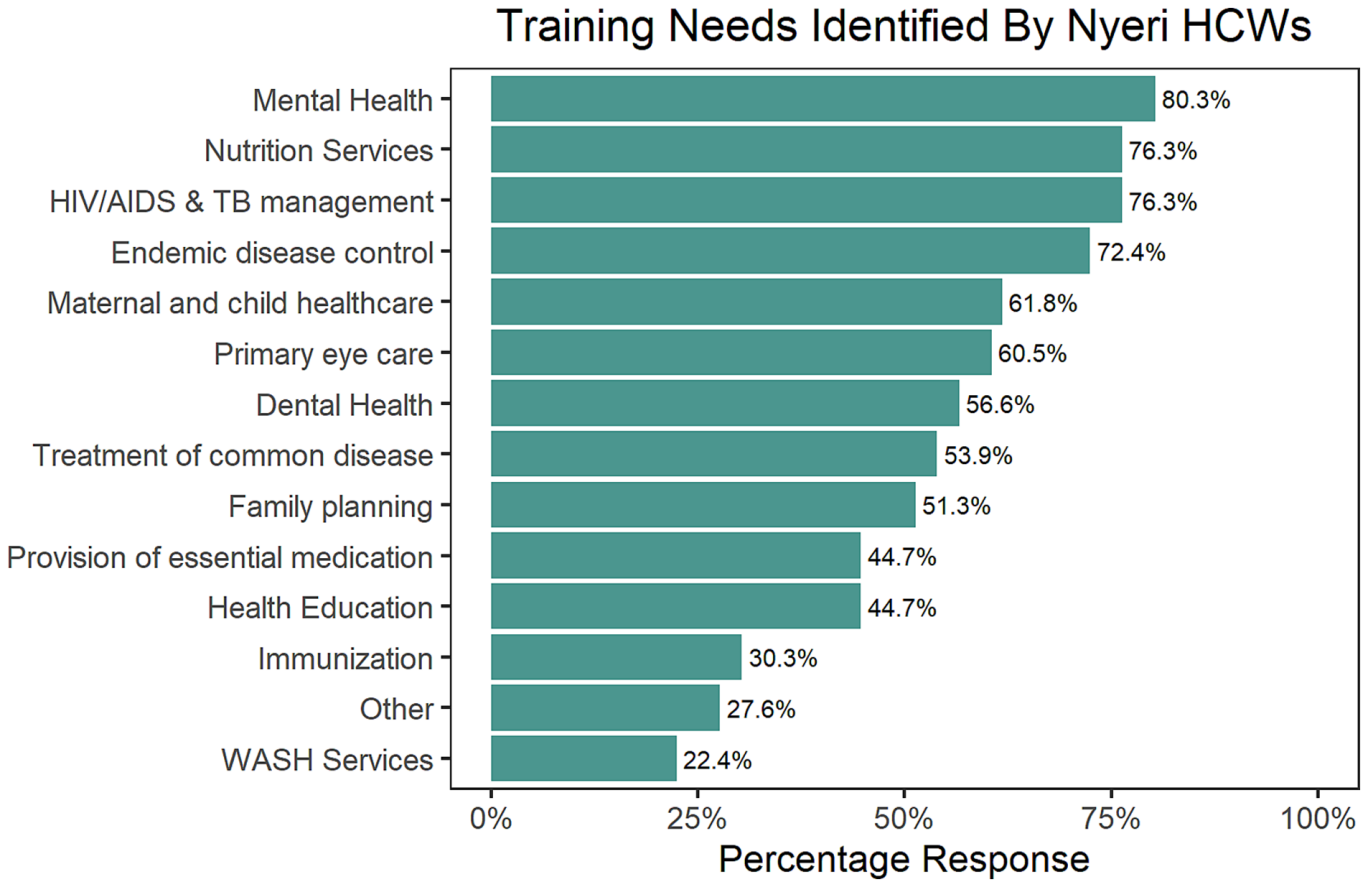
The training needs identified by Nakuru County HCWs, ranked in order of percentage frequency.

Responses from Nakuru HCWs revealed that HIV/AIDS and TB Management emerged as the top training priority, followed by maternal and newborn child healthcare and mental health training (Figure 4). Responses from Nyeri suggested that mental health training is a priority need. HIV/AIDs & TB management and a need for improved nutrition expertise featured prominently (Figure 5).

Key informants suggested that training in HIV/AIDs and TB Management should emphasize the latest treatment protocols, including how to manage drug-resistant strains. Infection control strategies, patient-centered counseling, and collaboration with specialized care programs were identified as essential components to enhance the overall quality of care for these complex conditions. For maternal healthcare, they suggested that continuous training in emergency obstetric and neonatal care, breastfeeding promotion, and recognizing high-risk pregnancies can further augment existing expertise and promote positive outcomes.

They also noted that mental health training should equip providers with the ability to recognize common mental disorders, administer basic psychological first aid, and understand referral pathways for specialized care. Reducing stigma and fostering mental health inclusion are crucial objectives for mental health training programs. They alluded that nutrition training should address malnutrition, provide individualized dietary guidance, and promote optimal health across all ages to strengthen nutritional expertise.

## 4 Discussion

This study examined the capacity of HCWs in Nyeri and Nakuru counties to provide healthcare services. Our analysis revealed that the workers in the two counties had a high capacity to provide primary healthcare. We observed high confidence in providing education on health problems, maternal and newborn child healthcare, family planning, water and sanitation, immunization, local endemic disease control, treatment of common illnesses, and the provision of essential medication. These findings are consistent with other relevant studies: Couper et al. reported that a larger proportion of mid-level health workers in Kenya felt that their basic training was adequate for their work and highlighted a need to improve the relevance of training to their work (17). In a study among HCWs in Uasin Gishu County, a large proportion passed a written examination on neonatal resuscitation, but none demonstrated all the necessary skills in a simulation assessment (18). These results correspond to other relevant studies. In a similar study conducted among HCWs in China, Myanmar, Indonesia, Thailand, Vietnam, Cambodia and Malaysia showed that the ability dimension of capacity was rated lowest while the knowledge dimension rate highest (19). This suggests that the actual performance of HCWs does not meet the PHC system needs. Gaps in skills needed to address the major causes of disease burden in these counties should be further studied to align the skillset of the HCWs to community needs.

We found areas of lower confidence levels, such as in HIV/AIDs & TB management; mental health, and dental health. This suggests a gap between the performance of the HCWs and the objectives of the healthcare system. Dental health is a major concern, indicating a potential lack of training or resources dedicated to oral health. This component is particularly influenced by cultural or local influences, such that some HCWs themselves had poor oral practices. A study in a district in India depicted a high proportion of HCWs using indigenous products instead of toothpaste and tooth brush, and also regular use of tobacco by chewing and smoking (20). This might call for contextualized training that addresses common cultural practices that harm dental health, especially in rural areas.

Mental health and HIV/AIDS & TB management also show a need for increased competency, specialized training, and support. Korhonen et al., found similar gaps in mental health literacy levels among HCWs in South Africa and Zambia, and found associations with a lack of formal mental health training (21). In addition, they found that nurses with less training had prejudiced and fearful perceptions of people with mental health. However, they found that mental health assessment scales experience promoted the HCWs’ literacy levels. These may be good tools to utilize in trainings.

Uncertainty in primary eye care suggests that strengthening expertise and resources availability could improve access to basic eye health services. A study on the capacity of HCWs to provide primary eye care in Northern Nigeria also found skill weaknesses and misalignment to guidelines, even though the HCWs had good knowledge of common eye diseases (22). Another similar study with participants from Kenya, Malawi and Tanzania showed that only 41% of those trained were able to perform visual acuity tests, which are a critical determinant of eye condition management. The same study showed that only 3% of Kenyan participants had refresher training (23). These findings call for an investigation of the primary eye care curricula. The low confidence ratings in these areas call for the attention of policymakers in the two counties.

In line with WHO recommendations (24), efforts should not only increase the number of healthcare workers but also boost their quality and relevance. There is a need to review the HCW training curriculum according to the disease burden of the populations served. Wilson et al., report the importance of training healthcare providers in the location of future work to equip them for the diverse working environments (25). This calls for better structured and supervised recruitment and placements, especially in rural areas.

Nakuru healthcare workers rated themselves better than their Nyeri counterparts in maternal and newborn child healthcare, local endemic disease control, appropriate treatment of common diseases and injuries, provision of essential basic medication, dental health, HIV/AIDs & TB management, and primary eye care. Nyeri HCWs had a greater median working experience than those in Nakuru. Continuous professional development and follow-up training among Nyeri HCWs is critical for improved performance. We also recommend the use of continuous professional development credits by Professional Boards for continued registration. Data suggests that the critical areas for improvement in Nyeri are primary eyecare and HIV/AIDs & TB management.

In our comparison between health facility levels, we found that the level 2 HCWs in Nakuru had a higher perceived capacity to provide family planning services, which is an ideal case. This means the population has ease of access to high-quality family planning services. This situation reduces the risk of discontinued use of contraceptives when still necessary (26). At the same time, we found a significant gap between the capacity of HCWs in level 4 and level 2 facilities to provide HIV/AIDs & TB management services. A previous study showed that residents of Nakuru county often chose HIV testing centers based on proximity and preferred either dedicated voluntary counseling and testing (VCT) facilities or a private doctor’s office for testing (27). Masini et al., also demonstrated the invaluable place of healthcare workers for directly observed therapy to increase TB treatment adherence (28). There is a need to build capacity for lower-level facilities to match the needs of people in the community.

For Nyeri County, we found a significant weakness in capacity at level 3 facilities for immunization against major diseases compared to the other two facilities. The greatest discrepancy was in dental healthcare, as the mean confidence ratings lay between slightly confident and neutral. The burden of oral health problems and unmet treatment needs among Kenyan adults is heavy (29). Dental practice remains at the periphery of PHC systems (30) because oral health training is mostly clinical. This calls for dental medical schools to determine ways of aligning their training to the principles of PHC. Similarly, there is a need for capacity building for mental health and primary eyecare at lower-level facilities.

### 4.1 Knowledge gaps and next steps

This paper provides insight to the stalled progress in primary healthcare system implementation in Kenya. The recommendations for additional training or retraining of HCWs for capacity building assume that adequate training would have an impact on performance. It is necessary to determine why the capacity levels are low specifically on the four additional components of primary health care included for the Kenyan context. There are studies that have shown a disconnect between the training that HCWs undergo and their actual performance, even under the influence of cultural practices and beliefs. A broad picture of the various barriers to progress in implementing PHC in Kenya is necessary for well-informed strategies.

There are studies that have conducted pre-training and post-training study designs to examine changes in patient outcomes after HCW education interventions in other countries that may be insightful for the Kenyan health system (31–33). It is noted that each of these studies used established programs for continuous medical education for diverse PHC components leading to significant performance improvements, but one in particular implemented a training-of-trainers for scaling. These are all options that can be leveraged for the Kenyan system.

With these in mind, the Kenyan Ministry of Health in partnership with development partners are positioned to address the gaps in knowledge and also the disconnect between knowledge and practice by: employing Continual Medical Education programs that engage high-fidelity simulation training; interactive case-based learning and participatory training; contextualizing the trainings to the different counties as the HCW demographics vary by age and experience; assessing the effectiveness of these programs on the basis of patient outcomes before and after the trainings; developing a training-of-trainers model to scale the delivery of high quality training programs and establishing a network for inter-county cooperation for diffusion of solutions among the counties. It is hoped that these steps can improve the capacity of HCWs for delivering PHC services in Kenya.

### 4.2 Strengths and limitations of the study

The purpose of our study was to assess the capacity of healthcare workers in Nyeri and Nakuru counties. We also obtained data on their training needs through the questionnaire, and through key informant interviews with both County Health Management Teams and Sub-County Health Management Team members. This study had limitations that must be considered while interpreting the results. To some extent, the relatively small sample size limits the representativeness of the HCW population per cadre and level of facility. Even when considering the different contexts of the two counties, they are relatively close geographically and more urbanized, limiting generalizability to other regions in the country. This study was also based on a self-reported questionnaire on reported capacity but does not assess the knowledge or practical skills of the HCWs. This calls for caution in interpreting perceptions due to reporting bias.

## 5 Conclusion

This study found a substantial capacity gap in several of the elements of PHC, especially in Nyeri County. Critical areas for intervention are HIV/AIDs & TB management and mental health training for both counties. Within the health systems, there is a need to strengthen the capacity of HCWs in lower-level health facilities to reduce the volume of referrals to secondary care facilities. Special attention should be given to mental health, dental health and primary eyecare in these counties. The government and development partners should facilitate practical training and align the curriculum to the elements of primary healthcare, while also building the capacity of lower-level facilities to provide primary healthcare.

## Data Availability

The raw data supporting the conclusions of this article will be made available by the authors, without undue reservation.
