# Supplementary material for: The capacity and training needs of primary health care workers in Nyeri and Nakuru counties of Kenya: a cross-sectional study
Source: Front Med (Lausanne). 2025 Jan 7;11:1466383. doi: 10.3389/fmed.2024.1466383 (PMC11753224; doi:10.3389/fmed.2024.1466383)
Supplement: Supplementary file 1 [file Table_1.DOCX]

### Project Thrive Baseline Assessment: KII Guide for County Officials & Amref Staff

**Introduction:**

Thank you for agreeing to participate in this interview. This project aims to assess the current state of primary healthcare in Nyeri and Nakuru Counties, Kenya, to establish a baseline for evaluating the impact of Project THRIVE. Your insights as a key stakeholder are crucial in understanding existing needs and challenges.

**Interview Questions:**

**Section 1: Service Availability**

1. What types of healthcare services are currently offered at your county's health facilities?

***Probe:***

- ***Education on health problems and how to prevent and control them***
- ***Development of adequate food supply and proper nutrition***
- ***Maternal and newborn child healthcare***
- ***Family planning***
- ***Adequate and safe water supply and basic sanitation***
- ***Immunizations against major infectious diseases, e.g. COVID***
- ***Local endemic disease control, e.g. Malaria***
- ***Appropriate treatment of common diseases and injuries***
- ***Provision of essential basic medication***
- ***Dental Health***
- ***Mental Health***
- ***HIV/AIDS & TB management***
- ***Primary eye care***

1. Are there any significant gaps or limitations?
2. What are basic diagnostics and treatment equipment available, e.g. BP machines and glucometers?

**Section 2: Accessibility and Utilization**

1. In a scale of 1 (very difficult) and 5 (very easy), how easy is it for residents in your county, particularly those in underserved communities, to access primary healthcare services?
2. What are the reasons for the above response?

***Probe:***

- ***If difficult/very difficult, what are the specific challenges preventing people from seeking healthcare when needed?***
- ***If easy/very easy, what are the specific enablers that prevent people from seeking healthcare when needed?***

**Section 3: Workforce Capacity**

1. In your perspective, what are the current skills and knowledge levels of healthcare workers in addressing common diseases and providing basic services?

***Probe:***

- ***Education on health problems and how to prevent and control them***
- ***Development of adequate food supply and proper nutrition***
- ***Maternal and newborn child healthcare***
- ***Family planning***
- ***Adequate and safe water supply and basic sanitation***
- ***Immunisation against major infectious diseases e.g. COVID***
- ***Local endemic disease control e.g. Malaria***
- ***Appropriate treatment of common diseases and injuries***
- ***Provision of essential basic medication***
- ***Dental Health***
- ***Mental Health***
- ***HIV/AIDS & TB management***
- ***Primary eye care***

1. Considering the current priorities and challenges in PHC delivery, what specific areas of training would most benefit healthcare workers?

**Section 4: Community Engagement and Information Sharing**

1. What community outreach programs or initiatives are currently in place to promote health education and awareness in your county?
2. How effectively do these programs reach residents and address their knowledge gaps regarding healthcare?

**Section 5: Data-Driven Decision Making**

1. How is data on primary healthcare needs and service utilisation currently collected and used by the county health department?
2. What are the limitations in how data is used to inform healthcare decisions?

**----------------------------Thank you again for your participation! ------------------------**
