# Supplementary material for: The capacity and training needs of primary health care workers in Nyeri and Nakuru counties of Kenya: a cross-sectional study
Source: Front Med (Lausanne). 2025 Jan 7;11:1466383. doi: 10.3389/fmed.2024.1466383 (PMC11753224; doi:10.3389/fmed.2024.1466383)
Supplement: Supplementary file 2 [file Table_2.DOCX]

### Project Thrive Baseline Assessment: Questionnaire for HCWS

**Introduction:**

Thank you for participating in this baseline assessment for Project THRIVE. This project aims to improve access to quality primary healthcare services in Nyeri and Nakuru Counties, Kenya. Your responses will help us understand the current state of healthcare delivery.

Your participation is voluntary, and all your responses will be kept confidential.

**Name of Interviewer**: _______________

**Date**: ___________________

**Section 1: Background Information**

| 1. County | 1. Nyeri 2. Nakuru |
| --- | --- |
| 1. Sub-county |  |
| 1. Name of facility |  |
| 1. Level of facility | 1. Level 4 2. Level 3 3. Level 2 |
| 1. Cadre | 1. Doctor 2. Clinic Officer 3. Nurse 4. Public Health Officer 5. Nutritionist 6. Other (specify) |
| 1. Duration worked in current role |  |
| 1. Years of experience in the current facility |  |
| 1. Number of healthcare workers currently working in the facility |  |
| 1. Which section of the Hospital are you working (Please check all that apply) | 1. OPD 2. Casualty/accident and Emergency 3. MHC 4. Pharmacy 5. Orthopedics 6. Maternity 7. Comprehensive care center 8. Lab & Blood Bank 9. Ophthalmology 10. In-patient 11. Operating theatre 12. Radiology 13. Dental 14. Pediatrics 15. Health Records 16. Physiotherapy 17. Public Health 18. Medical Engineering & Maintenance 19. Specialised clinics 20. Other (specify) |

**Section 2: PHC Service Availability**

**Instructions:** Please indicate (Yes/No) whether your facility currently offers the following services:

| **Description** | **Response** |
| --- | --- |
| 1. Health education | 1. Yes 2. No |
| 1. Nutritional services | 1. Yes 2. No |
| 1. Maternal and newborn child healthcare [*Focused Antenatal Care, Emergency Maternal Obstetric and Neonatal Care (EmONC), Newborn Care, Maternal Infant and young child feeding (MIYCF), Integrated Management of Childhood Illness (IMCI), Integrated Management of Acute Malnutrition (IMAM)]* | 1. Yes 2. No |
| 1. Family planning [*Long acting & Reversible methods of Family Planning, Cervical Cancer Screening, Sexual Gender Based Violence (SGBV)]* | 1. Yes 2. No |
| 1. Adequate and safe water supply and basic sanitation | 1. Yes 2. No |
| 1. Immunisation against major infectious diseases e.g. COVID | 1. Yes 2. No |
| 1. Local endemic disease control e.g. Malaria | 1. Yes 2. No |
| 1. Appropriate treatment of common diseases and injuries | 1. Yes 2. No |
| 1. Provision of essential basic medication | 1. Yes 2. No |
| 1. Dental Health | 1. Yes 2. No |
| 1. Mental Health | 1. Yes 2. No |
| 1. HIV/AIDS & TB management | 1. Yes 2. No |
| 1. Primary eye care | 1. Yes 2. No |

**Section 3: Training and Skills**

**Instructions:** Please rate your confidence level in performing the following tasks on a scale of 1 (Not Confident) to 5 (Very Confident).

| **Description** | **Rate** |
| --- | --- |
| 1. Education on health problems and how to prevent and control them. | 1 (Not Confident)  2 (Slightly Confident)  3 (Neutral)  4 (Confident)  5 (Very Confident) |
| 1. Development of adequate food supply and proper nutrition | 1 (Not Confident)  2 (Slightly Confident)  3 (Neutral)  4 (Confident)  5 (Very Confident) |
| 1. Maternal and newborn child healthcare [*Focused Antenatal Care, Emergency Maternal Obstetric and Neonatal Care (EmONC), Newborn Care, Maternal Infant and young child feeding (MIYCF), Integrated Management of Childhood Illness (IMCI), Integrated Management of Acute Malnutrition (IMAM)]* | 1 (Not Confident)  2 (Slightly Confident)  3 (Neutral)  4 (Confident)  5 (Very Confident) |
| 1. Family planning [*Long acting & Reversible methods of Family Planning, Cervical Cancer Screening, Sexual Gender Based Violence (SGBV)]* | 1 (Not Confident)  2 (Slightly Confident)  3 (Neutral)  4 (Confident)  5 (Very Confident) |
| 1. Adequate and safe water supply and basic sanitation | 1 (Not Confident)  2 (Slightly Confident)  3 (Neutral)  4 (Confident)  5 (Very Confident) |
| 1. Immunisation against major infectious diseases e.g. COVID | 1 (Not Confident)  2 (Slightly Confident)  3 (Neutral)  4 (Confident)  5 (Very Confident) |
| 1. Local endemic disease control e.g. Malaria | 1 (Not Confident)  2 (Slightly Confident)  3 (Neutral)  4 (Confident)  5 (Very Confident) |
| 1. Appropriate treatment of common diseases and injuries | 1 (Not Confident)  2 (Slightly Confident)  3 (Neutral)  4 (Confident)  5 (Very Confident) |
| 1. Provision of essential basic medication | 1 (Not Confident)  2 (Slightly Confident)  3 (Neutral)  4 (Confident)  5 (Very Confident) |
| 1. Dental Health | 1 (Not Confident)  2 (Slightly Confident)  3 (Neutral)  4 (Confident)  5 (Very Confident) |
| 1. Mental Health | 1 (Not Confident)  2 (Slightly Confident)  3 (Neutral)  4 (Confident)  5 (Very Confident) |
| 1. HIV/AIDS & TB management | 1 (Not Confident)  2 (Slightly Confident)  3 (Neutral)  4 (Confident)  5 (Very Confident) |
| 1. Primary eye care | 1 (Not Confident)  2 (Slightly Confident)  3 (Neutral)  4 (Confident)   1. (Very Confident) |

1. Please select the areas most relevant to your needs. You can also mention any other areas you feel require attention in the space provided below:

**Section 4: Collaboration and Communication**

**Instructions:** Please rate your level of agreement with the following statements using a scale of 1 (strongly disagree) to 5 (strongly agree).

| **Description** | **Rate** |
| --- | --- |
| 1. There are clear guidelines for referral processes between health facilities and referral hospitals | 1 (Strongly disagree)  2 (Disagree)  3 (Neutral)  4 (Agree)  5 (strongly Agree) |
| 1. Communication channels between health facilities and referral hospitals are efficient and reliable | 1 (Strongly disagree)  2 (Disagree)  3 (Neutral)  4 (Agree)  5 (strongly Agree) |
| 1. There is a system in place for timely feedback on referred patients from referral hospitals to health facilities | 1 (Strongly disagree)  2 (Disagree)  3 (Neutral)  4 (Agree)  5 (strongly Agree) |
| 1. Regular meetings or training sessions are conducted to improve collaboration between healthcare facilities and sub/county management teams | 1 (Strongly disagree)  2 (Disagree)  3 (Neutral)  4 (Agree)  5 (strongly Agree) |

**Section 5: Data Collection**

**Instructions:** Please answer the following questions to the best of your ability.

| **Description** | **Rate** |
| --- | --- |
| 1. Are you involved in any data collection activities related to primary healthcare services at your facility? | Yes  No  Not sure |
| 1. Do you currently have any standardized data collection tools (registers) for PHC data collection? | Yes  No  Not sure |

**----------------------------Thank you again for your participation! ------------------------**
